# Genome-wide association study of aphid abundance highlights a locus affecting plant growth and flowering in *Arabidopsis thaliana*

**DOI:** 10.1098/rsos.230399

**Published:** 2023-08-23

**Authors:** Chongmeng Xu, Yasuhiro Sato, Misako Yamazaki, Marcel Brasser, Matthew A. Barbour, Jordi Bascompte, Kentaro K. Shimizu

**Affiliations:** ^1^ Department of Evolutionary Biology and Environmental Studies, University of Zurich, Winterthurerstrasse 190, 8057 Zurich, Switzerland; ^2^ Research Institute for Food and Agriculture, Ryukoku University, Yokotani 1-5, Seta Oe-cho, Otsu, Shiga 520-2194, Japan; ^3^ Départemente de Biologie, Université de Sherbrooke, 2500 boulevard de l'Université, Sherbrooke, Quebec, Canada J1K 2R1; ^4^ Kihara Institute for Biological Research, Yokohama City University, Maioka 641-12, Totsuka-ward, Yokohama 244-0813, Japan

**Keywords:** genome-wide association study, plant–insect interaction, herbivory

## Abstract

Plant life-history traits, such as size and flowering, contribute to shaping variation in herbivore abundance. Although plant genes involved in physical and chemical traits have been well studied, less is known about the loci linking plant life-history traits and herbivore abundance. Here, we conducted a genome-wide association study (GWAS) of aphid abundance in a field population of *Arabidopsis thaliana*. This GWAS of aphid abundance detected a relatively rare but significant variant on the third chromosome of *A. thaliana*, which was also suggestively but non-significantly associated with the presence or absence of inflorescence. Out of candidate genes near this significant variant, a mutant of a ribosomal gene (AT3G13882) exhibited slower growth and later flowering than a wild type under laboratory conditions. A no-choice assay with the turnip aphid, *Lipaphis erysimi*, found that aphids were unable to successfully establish on the mutant. Our GWAS of aphid abundance unexpectedly found a locus affecting plant growth and flowering.

## 1. Introduction

Plants are consumed by herbivores throughout their life cycles in natural environments. While it is well recognized that chemical and physical traits affect herbivore abundance on individual plants [1–4], plant life-history traits also account for variation in herbivore abundance in field environments [5–7]. For example, plant size, flowering and other visible traits are known to affect the probability of colonization by herbivores [8–11]. By focusing on genetic variation within a plant species, several studies have shown that plant life-history traits shape heritable variation in herbivore abundance and community composition [4,12,13]. Yet, genes or loci underlying plant life-history traits and herbivore abundance have not been identified until recently [14,15].

Genome-wide association study (GWAS) is an effective way to dissect the genetic architecture of ecologically important traits [14,16]. GWAS provides a hypothesis-free approach to identify novel genes from natural phenotypic variation through associations between single nucleotide polymorphisms (SNPs) and traits [17,18]. Recent studies have shown that controlled laboratory conditions are unlikely to reflect outdoor environments where interspecific interactions typically occur [19,20]. This fact emphasizes the importance of *in natura* studies of functional genes [21–25]. To achieve this goal, it is important to conduct GWAS under field conditions.

*Arabidopsis thaliana* is a model plant species distributed and naturalized around the world. While *A. thaliana* usually blooms in spring after over-wintering, some cohorts have overlapping life cycles from spring to autumn [25–27]. When *A. thaliana* plants emerge from late spring to early summer, they are threatened by various herbivores [4,28]. Among the diverse insect herbivores, aphids are known to be the major herbivores occurring across the natural distribution range of *A. thaliana* [29]. Because aphids often suck phloem sap from leaf veins and inflorescences, we assumed that plant life-history traits may play a role in harbouring aphids under field conditions.

To reveal the genetic architecture of aphid abundance, we combined GWAS with mutant analysis in *A. thaliana*. We first conducted GWAS of aphid abundance on 196 *A. thaliana* accessions grown at a field site in Zurich, Switzerland. To further test candidate genes, we cultivated and released the turnip aphid *Lipaphis erysimi* on *A. thaliana* mutants.

## 2. Material and methods

### 2.1. Field genome-wide association study

#### 2.1.1. Plant genotypes

We obtained *Arabidopsis thaliana* genotypes that were selfed and maintained as inbred lines, called ‘accessions’, from the Arabidopsis Biological Resource Center (https://abrc.osu.edu/). We used the same set of 196 *A. thaliana* accessions as in a previous study [30] except for two trichome mutants and an ungenotyped accession. All accessions were genotyped in the RegMap [31] and 1001 Genomes [32] projects. The list of accessions and phenotypes measured in this study is available in electronic supplementary material, table S1.

#### 2.1.2. Field experiment

To observe aphids in a simulated late cohort, we exposed *A. thaliana* accessions to a field environment from 4 to 25 July 2018. This field experiment was conducted in Zurich, Switzerland, to use a field site within a native distribution range of *A. thaliana*. To maintain all accessions in the rosette stage at the start of the field experiment, we initially cultivated *A. thaliana* in a laboratory under a short-day condition (8 h light/16 h dark cycle at 20°C). Seeds were sown on 33 mm diameter Jiffy-seven pots and stratified under a constant dark 4°C condition for a week. Seedlings were cultivated in a growth chamber for six weeks under the short-day condition. Plants grown on the Jiffy-seven pots were then planted in a new plastic pot filled with mixed soils of agricultural composts (Profi Substrat Classic CL ED73, Einheitserde Co.) and perlites with a compost : perlite ratio = 3 : 1 litter volume. Eight replicates of the 196 accessions were then transferred to the outdoor garden at the University of Zurich-Irchel (47°23′ N, 8°33′ E). Aphids were identified and counted by a single observer (Y.S.) every two or three days. The two species of specialist aphids, *Lipaphis erysimi* and *Brevicoryne brassicae*, were identified based on the presence or absence of waxy compounds on their abdomens. These two species could be distinguished from the generalist aphid *Myzus persicae* based on the length of the cornicules, though *M. persicae* did not occur during the present field experiment. To examine whether the aphid abundance differed between plants with and without inflorescence, we also recorded the presence or absence of bolting two weeks after the start of the field experiment. The field experiment lasted three weeks after transplanting until early-flowering accessions of *A. thaliana* started terminating their life. Since 38% of plants initiated bolting in the field (see Results) and late-flowering accessions did not bloom unless vernalized [33], longer experiments were difficult due to the short life span of flowered *A. thaliana*.

#### 2.1.3. Data analysis

All GWAS analyses were performed using the GWA-portal (https://gwas.gmi.oeaw.ac.at) [34]. The imputed full-sequence dataset [34] was used as SNP data for the 196 accessions, which provided combined SNP data imputed between 250k SNP chip genotyping by the RegMap project [31] and high-throughput sequencing by the 1001 Genome Project [32]. Pseudo-heritability *h*^2^ [34] was calculated for the target phenotype before association mapping. Accelerated mixed models [34] were used for association mapping with a correction of the kinship structure. After association mapping, candidate genes were searched within *ca* 5 kb near a focal SNP. Genome sequences of the natural accessions were checked using the 1001 genome browser (http://signal.salk.edu/atg1001/3.0/gebrowser.php). To inspect organ-specific expression levels of candidate genes, we referred to Klepikova Arabidopsis Atlas [35] via the Arabidopsis Information Resource (https://www.arabidopsis.org/). The GWAS HitMap of AraGWAS Catalog (https://aragwas.1001genomes.org/#/map) [36] was also used to determine whether the top-scoring SNP was associated with other reported traits.

We analysed aphid abundance and two additional traits as the target phenotype in the GWA-portal. Aphid abundance was quantified as the maximum number of aphids, which included *Lipaphis erysimi* and *Brevicoryne brassicae* (see Results), observed on a plant during the experiment. The number of aphids was then ln(*x* + 1)-transformed to improve normality. We also analysed the presence (1) or absence (0) of bolting as a representative trait of flowering to examine its overlap with the top-scoring SNP of aphid abundance. To quantify the extent to which the exclusion of bolting effects weakened the top-scoring SNPs of aphid abundance, we also performed GWAS using residuals of the aphid abundance as a target trait. These residuals were obtained by regressing aphid abundance on the presence (1) or absence (0) of bolting using a standard linear model.

### 2.2. Mutant analysis

#### 2.2.1. *Arabidopsis thaliana* mutants

Transfer-DNA (T-DNA) sequence-indexed lines of *A. thaliana* were obtained from the Nottingham Arabidopsis Stock Centre (NASC) (https://arabidopsis.info/). In addition to Columbia-0 (Col-0, NASC Accession ID: N70000) wild type, we ordered four mutant lines for a ribosomal gene (AT3G13882) (electronic supplementary material, table S2). These original mutants were backcrossed with the Col-0 wild type three times.

Following the instructions [37], we examined the insertion site by polymerase chain reaction (PCR) amplification and Sanger sequencing; and gene expression levels by semi-quantitative reverse transcription and PCR (sqRT-PCR). To confirm the T-DNA insertion site of SALK_039481, we extracted DNA from leaves using the CTAB method. Using the primers shown in electronic supplementary material, tables S2 and S3, we amplified DNA by PCR as follows: 2 min at 95°C; 35 cycles of 15 s at 95°C, 30 s at 55°C, 1.5 min at 72°C; and a final extension step of 3 min at 72°C. The PCR product was sequenced by Sanger sequencing to confirm the insertion site.

For sqRT-PCR, we extracted RNA from the leaves using an RNeasy kit (Qiagen: catalogue no. 74181) and purified RNA using a DNA-free kit (Ambion: catalogue no. AM1906). RNA concentration was measured using a Qubit spectrophotometer (Invitrogen: catalogue no. Q10211). cDNA was obtained using a High-Capacity RNA-to-cDNA kit (Applied Biosystems: catalogue no. 4387406) from 500 ng of the total RNA. Using the primers shown in tables S3 and S4, we amplified cDNA with PCR as follows: 3 min at 95°C; 28 cycles of 15 s at 95°C, 30 s at 55°C, 1 min at 72°C; and a final extension step of 5 min at 72°C. Gel electrophoresis was performed on 1% agarose gel at 120 V for 60 min. The PCR products were visualized using a UV trans-illuminator system.

We found that one of the four lines, SALK_039481 (NASC Accession ID: N670586), indeed had a T-DNA insertion in an exon of one of the two splice variants (electronic supplementary material, figure S1) and reduced expression levels of AT3G13882 (electronic supplementary material, figure S2), suggesting that the insertion disrupted this gene. In the other three lines, the insertion was not found or a low germination rate prevented further experiments.

### 2.3. Laboratory experiments

To observe plant growth, we cultivated 10 replicates of the ribosomal gene mutant and the Col-0 wild type under long-day conditions (16 h light/8 h dark cycle at 22°C/20°C) (electronic supplementary material, figure S3). Seeds were sown on a 294 cm^3^ (= 7 *×* 7 *×* 6 cm^3^) pot filled with agricultural composts (Profi Substrat Classic CL ED73), and stratified at 4°C under a constant dark condition for a week. Stratified seeds were then transferred to long-day conditions. Seedlings were grown for 20 days. Rosette diameter (cm) was recorded as an index of plant size before aphids were released, as described below.

To test whether aphids could establish a colony on the mutant plants, we released the turnip aphid *L. erysimi* on the wild type and mutant *A. thaliana* plants used in the growth experiment described above (electronic supplementary material, figure S3). The potted plants grown for 20 days were separately enclosed with a mesh net. Five wingless adult female aphids were released on each plant. The experimental aphids were derived from a source population established by a previous study [15]. Enclosed plants were incubated under long-day conditions. The number of aphids per plant was counted by eye 3, 7, 10 and 14 days after the release of aphids. We did not count the aphids that escaped outside the area of the plant. Flowering time was defined as the number of days to flowering and was recorded until flowering.

### 2.4. Data analysis

We used linear mixed models (LMMs) or generalized linear mixed models (GLMMs) to test the phenotypic differences between the mutant and Col-0 wild type. Plant size and flowering time were analysed using LMMs that assumed Gaussian errors. The number of aphids, i.e. the count response, was analysed using GLMMs with a Poisson error structure and a log link function. Paired positions of a mutant and wild type plant were incorporated as a random effect to consider spatial heterogeneity within a growth chamber environment. An analysis of deviance with a *F*-test was used to test the significance of the mutant versus wild type (‘d.f. 1’ = 1) against phenotypic variation within the random effect of the paired positions (‘d.f. 2’ = 10 pairs minus 1 fixed effect = 9). To examine the effects of plant size and flowering time on the number of aphids, we also performed the same GLMM analysis with plant size or flowering time included as a log-offset term. All statistical analyses were performed using R version 4.0.3 [38]. For LMM and GLMM, we used the lmer and glmer functions implemented in the lme4 package [39].

## 3. Results

### 3.1. Field genome-wide association study of the aphid abundance

To monitor aphid abundance and visible plant traits, we transplanted 196 *A. thaliana* accessions in the field in Zurich within a native distribution range of *A. thaliana*. At the time of transplantation, all plants were at the rosette stage, i.e. no bolting occurred. After two weeks, 38% of the individual plants initiated bolting, i.e. inflorescence was observed. The main herbivores were the two species of specialist aphids, *Lipaphis erysimi* and *Brevicoryne brassicae*. The aphid abundance was higher on bolted accessions than on non-bolted accessions (non-bolted and bolted plants = average 0.59 and 2.07 aphids, respectively; Welch's *t*-test, *t* = −21.9, d.f. = 941.2, *p* < 10^−15^), suggesting that the plant life cycle might be associated with the plants' capacity to avoid aphids. In addition, we distinguished the abundance of winged and wingless aphids to infer the colonization process of aphids on *A. thaliana*. Winged and wingless aphids were observed at the rosette stage at the first monitoring after transplantation, but many of these aphids did not establish a colony in subsequent monitoring (the days between 7 and 10 July 2018; electronic supplementary material, figure S4). This observation suggests that colonized aphids do not always establish a colony and thus the success of colony establishment also depends on the presence of inflorescence after colonization.

**Figure 1.**
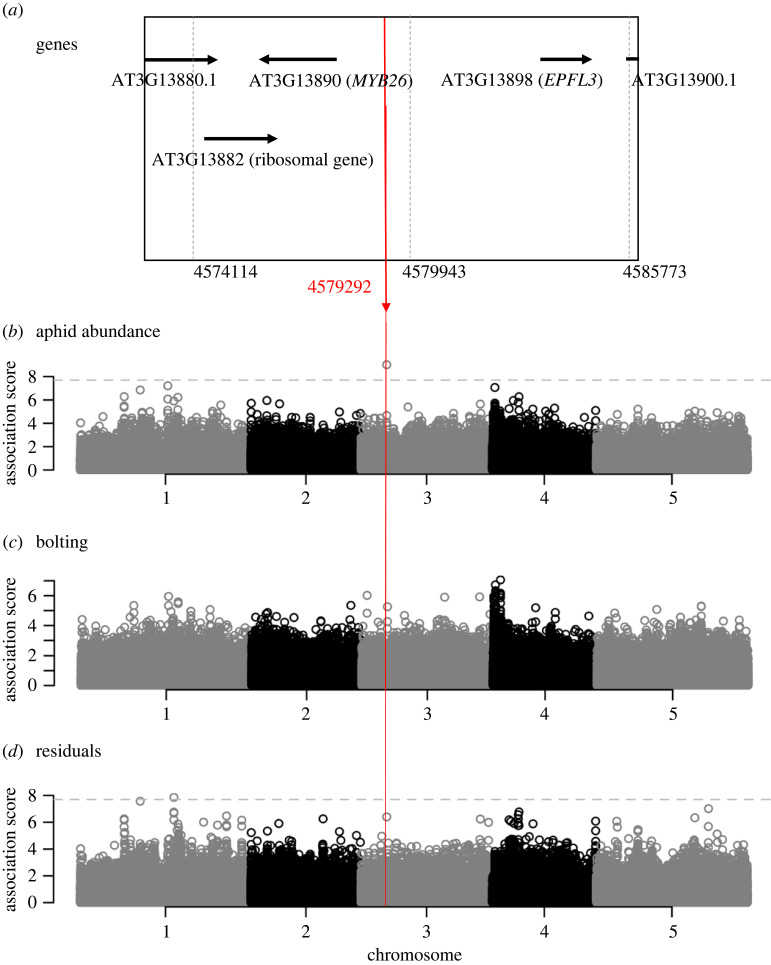
Genome-wide association study of aphid abundance on 196 *Arabidopsis thaliana* accessions grown in the field. (*a*) A genomic region within *ca* 5 kbp from the top-scoring SNP at Chr3-4579292 displays the position of candidate genes. Only the longest splice variant (black horizontal arrow) is shown for each gene. (*b–d*) Manhattan plots showing the association score of −log_10_(*p*) for aphid abundance (*b*), presence of bolting (*c*), and residuals of aphid abundance corrected by bolting (*d*) across five chromosomes of *A. thaliana* with a minor allele frequency (MAF) cut-off of 0.025. The horizontal dashed line indicates the genome-wide Bonferroni threshold at *p* = 0.05. The vertical red line highlights the position of Chr3-4579292.

To reveal the genetic architecture underlying variation in aphid abundance, we calculated heritability and then performed association mapping. Aphid abundance had high heritability among the plant accessions (*h*^2^ = 0.7), indicating genetic control of this trait. Our mapping also detected a significant SNP in an intergenic region above the genome-wide Bonferroni threshold (Chr3-4579292, *p* < 10^−8^, MAF = 0.026; figure 1*b*; see also electronic supplementary material, figure S5a, for quantile–quantile plots). This top-scoring SNP was also associated with the bolting to a non-significant but suggestive extent (−log_10_(*p*) = 5.26; figure 1*c*; electronic supplementary material, figure S5b). When we adjusted for the effects of bolting on aphid abundance (figure 1*d*; electronic supplementary material, figure S5c), the association of Chr3-4579292 with aphid abundance became weaker and less than the Bonferroni threshold, but remained at −log_10_(*p*) = 6.40. These results suggest the partial contribution of the bolting to shaping the significant association between the Chr3-4579292 SNP and aphid abundance, but a prominent association remained unexplained by bolting. According to the SNP viewer of the GWA-portal [34], the number of major and rare variants was 1956 and 73 at Chr3-4579292 among all the 2029 accessions registered in the portal site, where the relatively rare variant (less than 5% but more than 1% in MAF) was sporadically distributed among countries. Bolting and flowering time represent similar traits, and indeed we found the same broad peak on the top of the fourth chromosome, as reported by a previous GWAS of flowering time [40]. We did not find any other significant GWAS hits for Chr3-4579292 in the GWAS HitMap database [36] possibly because this rare variant might have been overlooked. GWAS analyses suggested that the SNP at Chr3-4579292 was significantly associated with aphid abundance through its potential influence on flowering.

To narrow down candidate genes, we further focused on the genomic region near the significant SNP at Chr3-4579292. Five out of the 196 accessions carried a rare variant that increased aphid abundance, while the other accessions had a major variant (electronic supplementary material, figure S6a). Genome sequences of four of the five rare accessions are available in the 1001 Genome Project [32], where three of the four available accessions, i.e. An-1, Kin-0, and Lm-2, shared similar patterns near Chr3-4579292 but differed from major accessions (electronic supplementary material, figure S6b). This additional evidence suggests that rare accessions carrying more aphids had consistent patterns near Chr3-4579292. Three candidate genes were located nearest to this SNP at Chr3-4579292 (figure 1*a*), encompassing a putative ribosomal gene (AT3G13882) that is homologous to a ribosome protein L34 gene (RPL34) [41], *EPIDERMAL PATTERNING FACTOR LIKE 3* (*EPFL3*: AT3G13898), and *MYB26*. Out of these three genes, the ribosomal gene (AT3G13882) is known to be highly expressed in vegetative organs such as leaves [35]. The other two genes, *EPFL3* and *MYB26*, are highly expressed only in reproductive organs such as anthers or pistils [35]. Because aphids were unlikely to suck saps from anthers and pistils, we focused on the ribosomal gene (AT3G13882) for further investigation.

### 3.2. Mutant plant growth and aphid colony establishment in the laboratory

**Figure 2.**
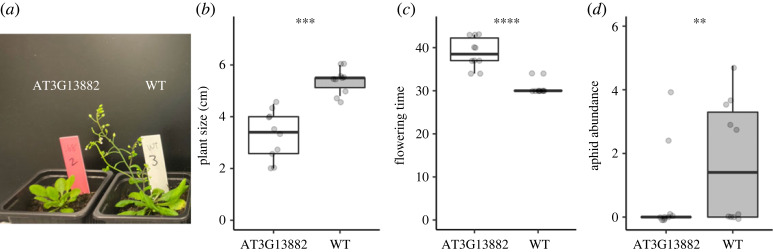
Photograph (*a*), plant size (*b*), flowering time (*c*) and aphid abundance (*d*) of the Col-0 wild type (WT) and ribosomal gene mutant (AT3G13882) of *Arabidopsis thaliana* under laboratory conditions. Flowering time and aphid abundance represent the number of days to flowering and log_2_(no. of aphids + 1), respectively. Asterisks indicate statistical significance with generalized linear mixed models; **p < 0.01; ***p < 0.001; ****p < 0.001. Boxes: median with upper and lower quartiles; whiskers: 1.5 *×* inter-quartile range.

To examine the visible phenotypes of the ribosomal gene mutant (AT3G13882), we compared the growth and flowering time of this mutant with those of Col-0 wild type. After 20 days of growth, the AT3G13882 mutant was significantly smaller than the wild type (*F*_1,9_ = 42.1, *p* = 0.00011; figure 2*a*,*b*). The flowering time of the AT3G13882 mutant was also significantly later than that of the wild type (*F*_1,9_ = 48.8, *p* < 0.0001; figure 2*a*,*c*). The slower growth and delayed flowering of the ribosomal gene mutant led us to test whether the delayed growth could prevent the establishment of aphid colonies after colonization.

To examine colony establishment after aphid colonization, we released wingless individuals of *Lipaphis erysimi* on 20-day-old rosette plants of the ribosomal gene mutant (AT3G13882) and wild type. We observed a reduced number of aphids on the AT3G13882 mutant compared to the wild type at 7, 10 and 14 days after the release of aphids (*F*_1,9_ = 19.3, *p* = 0.0017 at 7 days; figure 2*d*; see also electronic supplementary material, figure S7, for results at 10 and 14 days; electronic supplementary material, table S5), suggesting that delayed growth of the host negatively affected aphid colony establishment. We also incorporated plant size or flowering time as an offset term in the GLMMs to examine their confounding influence on aphid abundance. When the plant size was offset, the number of aphids differed less significantly between the wild type and mutant plants (*F*_1,9_ = 6.9, *p* = 0.027 at 7 days; see also electronic supplementary material, table S5). When the flowering time was offset, the number of aphids differed more significantly between the wild type and mutant plants (*F*_1,9_ = 40.78, *p* < 0.001 at 7 days; see also electronic supplementary material, table S5). These additional analyses suggested that delayed growth rather than delayed flowering contributed more to the unsuccessful establishment of aphid colonies, though these two phenotypes were highly correlated (Pearson's correction coefficient, *r* = −0.913, *p* < 10^−7^). However, the significant difference in aphid abundance between the wild type and mutant remained even after size or flowering was offset (electronic supplementary material, table S5), indicating the relevance of other traits to aphid colonization.

## 4. Discussion

Guided by the field GWAS of aphid abundance, we found that a mutant plant of ribosomal gene AT3G13882 exhibited slower growth and was less likely to harbour aphids in *A. thaliana*. While ribosomal genes have long been considered housekeeping genes of the protein synthesis machinery, mutants of ribosome-related genes exhibit a wide variety of growth and reproductive phenotypes. For example, previous studies have reported a reduction in leaf cell number [42], reduced root length [43], and a reduction in the number of pollen [18,44] regarding ribosomal gene mutations. We should note, however, that further studies on natural variants responsible for delayed growth and reduced aphid abundance are necessary to validate its importance in the field. Because linkage disequilibrium is common in the genome of *A. thaliana* [45], genes nearby the top-scoring SNP are equally possible to be causal. Multiple alleles of the AT3G13882 gene are thus needed to provide strong evidence of delayed growth phenotypes. In the studies of a ribosomal gene *REDUCED POLLEN NUMBER1* (*RDP1*), null mutants showed a pleiotropic effect on plant growth and pollen number in *A. thaliana* [18,44]. Natural alleles of *RDP1* can alleviate pleiotropic growth defects [18]. In our study, other growth-related genes or other mutations of AT3G13882 might have reduced aphid abundance. Because transgenic approaches may not be effective to identify mutation sites affecting quantitative traits, further experimental tests, such as quantitative complementation and genome editing [18,44], are needed to study natural causal variants that alter aphid abundance through delayed growth.

In the present study, we found limited evidence for the beneficial roles of delayed growth in escape from herbivores. Despite the significant difference in the initial colonization of aphids, two individuals with a mutation on the AT3G13882 gene were harboured by aphids in the laboratory (figure 2*d*). The flowering time of these two individuals was not hindered (the initial plant size of 4.0 cm and 34 days until flowering in figure 2*b*,*c*), suggesting that aphid colonization exerted little effect on plant reproduction. The advantage of slower growth may be cancelled if aphids can successfully colonize slow-growth accessions in the later period and reach an abundance similar to that of fast-growing accessions. Although such long-term effects of aphid colonization on plant fitness are difficult to evaluate using short-lived annual *A. thaliana*, this aspect could be tested with recurrent establishment of seasonal cohorts in *A. thaliana* [46]. Further studies on fitness consequences, in addition to the identification of natural causal variants, are needed to test whether delayed growth is adaptive as an active strategy of plant defence.

Although our GWAS of aphid abundance detected a significant variant that was also suggestively but non-significantly associated with the bolting, these effects of the bolting on aphid abundance were not separable from other traits affecting herbivore abundance. In the field data, the significant SNP became non-significant but remained suggestive even after adjusting for the effects of the bolting on aphid abundance (figure 1*d*). Laboratory experiments also showed that significant differences in aphid abundance between the wild type and mutant plants remained even after the effects of plant size or flowering were offset. These results suggest the pleiotropic contributions of other traits to aphid abundance. Specifically, we found fewer aphids on slow-growth mutants even under no-choice conditions in the laboratory (figure 2*d*), suggesting that traits co-varying with plant growth or flowering, such as plant nutritional values [47] and secondary metabolites [48], were likely involved in the aphid colonization. Thus, our results should be carefully interpreted with respect to other traits responsible for reduced aphid abundance.

In summary, we found a novel quantitative trait locus related to plant growth and aphid abundance. While previous field studies have illustrated *in natura* roles of well-studied functional genes in chemical resistance (e.g. *LOX*s in *Nicotiana attenuata* [2,3]) and physical resistance (*GLABRA1* in *A. thaliana* [4]) to herbivores, our GWAS of aphid abundance unexpectedly detected a locus related to plant growth rather than resistance. Nonetheless, these studies suggest that plant genetic variation governs herbivore abundance and community structure [4,12–14]. A keystone gene shaping ecological communities has recently been identified [15]. Barbour *et al*. [15] have experimentally shown that pleiotropic effects of a glucosinolate biosynthesis gene *AOP2* on plant growth alter *A. thaliana*'s capacity to harbour aphids and their parasitoids. As aphids and aphidophagous insects are widespread across terrestrial ecosystems [49,50], future studies may reveal the cascading effects of delayed plant growth on food webs.

## Data Availability

The data and codes are available at the GitHub repository (https://github.com/yassato/AraAphidGWAS) and the published version is deposited on Dryad (https://doi.org/10.5061/dryad.pg4f4qrvg) [51]. The supplementary figures and tables are provided in electronic supplementary material [52].
